# Investigating Painkiller Use in Amateur Football: A Coach’s Perspective

**DOI:** 10.3390/jpm14091003

**Published:** 2024-09-20

**Authors:** Andreas Kopf, Werner Krutsch, Dominik Szymski, Johannes Weber, Volker Alt, Hermann Josef Bail, Niklas Engel, Johannes Rüther, Lorenz Huber

**Affiliations:** 1 Department of Orthopaedics and Traumatology, Paracelsus Medical University, Breslauer Straße 201, 90471 Nuremberg, Germany; andreas.kopf@klinikum-nuernberg.de (A.K.); niklas.engel@klinikum-nuernberg.de (N.E.);; 2 Department of Trauma Surgery, University Medical Centre Regensburg, Franz-Josef-Strauss Allee 11, 93053 Regensburg, Germany; 3 FIFA Medical Centre of Excellence, University Medical Centre Regensburg, 93053 Regensburg, Germany; 4 SportDocsFranken, 90473 Nuernberg, Germany

**Keywords:** football, soccer, amateur sports, medication, recreational, painkiller use

## Abstract

Background/Objectives: Painkiller use in amateur sports and, especially, in football is increasingly being discussed, but the scientific data on this field are very limited. Therefore, the aim of this study was to investigate the prevalence of painkiller use in amateur football from the perspective of coaches, as well as to determine how and to which extent coaches can influence their teams in terms of painkiller use and prevention. Methods: Using an online questionnaire, a cross-sectional analysis of painkiller use in German amateur football from the 4th league to the lowest amateur classes was carried out from the perspective of team coaches. A total of 628 participants were contacted, and 400 (63.7%) completed the questionnaire completely and were therefore included in the evaluation. Results: Of the 400 participating team coaches in amateur football, 369 (92.3%) were male and 31 (7.7%) were female. The coaches reported that 36.2% (SD = 29.1) of their players have used painkillers at some point due to football-related pain in their career. The majority of coaches believed that the use of painkillers is not compatible with competition (74%), and even more believed that it is not compatible with football training (90.8%). Furthermore, 56.2% of the coaches themselves had already taken painkillers in their own football career for football-related pain, and 64% had already bought over-the-counter painkillers without a prescription. The use of painkillers increased in the higher playing levels. The availability of painkillers in first aid kits was reported by around 60%, but they were reported as freely accessible in the dressing room by only 10% of the coaches. Conclusions: This is the first study to describe the painkiller use in amateur football from the perspective of coaches. The prevalence of painkiller use in this study was found to be significantly lower than what is indicated in the data from the existing literature. The majority of coaches see the use of painkillers during games and training as incompatible, even though there is a large proportion of coaches who have already bought over-the-counter painkillers for football-related pain. As the first scientific analysis of team coaches, this study provides fundamental data for the prevention of excessive painkiller use in amateur football.

## 1. Introduction

The use of painkillers in football has received great public and scientific interest in recent years. Although the majority of the scientific data come from professional sports, there are also studies on the prevalence of painkiller use in amateur sports [1,2]. In professional sports, the use of painkillers has been described as having a higher prevalence than in the general population, regardless of the type of sport [3]. Most studies on painkiller use in sports focus on the major events with many participants. However, individual events like marathons often show high rates of painkiller use among non-professional athletes, as shown by quantitative studies [4,5].

In football, previous research has systematically collected data that depicts the use of medication at the FIFA World Cups from 2002 to 2018. These studies showed that more than half (55%) of the players took NSAIDs over the course of the tournament, with a enduringly constant prevalence of use over the years. They also showed that around 30% of players take NSAIDs directly before a game [6,7,8,9]. It is not known whether the use of painkillers was medically indicated or was due to dependence for treating pain or injuries. In addition, it was not described whether the medication was self-administered or prescribed by team doctors. Individual studies in professional football that have investigated the prevalence of painkiller use over an entire season have shown an even higher prevalence of 66% to over 90% [10,11]. However, the results of a meta-analysis involving around 40,000 data sets suggest that these findings cannot simply be transferred to amateur sports as it was shown that non-competitive athletes have a significantly lower use of painkillers compared to professional athletes [12]. The high intensity in professional football, especially at major tournaments such as the World Cup, raises doubts about whether the data collected applies to amateur football. Leyk et al. (2023) highlighted this research deficit in their review and called for the collection of extensive data on amateur athletes, especially footballers (who have come under particular public criticism) [3]. 

The goal of this study was, therefore, to analyse the painkiller use in national amateur football from the perspective of responsible coaches. This includes both the painkiller prevalence from the coaches’ perspective and the role of coaches in relation to painkiller use. The coaches’ attitude towards the compatibility of painkiller use in football, as well as their knowledge of the effects and side effects of painkillers, were also investigated. The hypothesis of this study was to understand whether the prevalence and patterns of painkiller use in amateur football are comparable to those found in other amateur sports or professional football cohorts investigated in previously published studies. Additionally, this study aimed to identify potential preventive strategies to reduce painkiller use in amateur football.

## 2. Methods

In this prospective cohort study, an online questionnaire was specifically designed for team coaches of German amateur football teams. The analysed football teams belong to the regional Football Association in Germany, the Bavarian FA, which includes more than 1.6 million football players and more than 4500 football clubs. The study population includes amateur teams from the highest amateur playing level (4th league) to the lowest playing levels and veteran football teams. All of the invited team coaches of the attending football clubs were able to complete the questionnaire anonymously from January 2022 to July 2022. The coaches were contacted via the Bavarian Football Association (BFV) and its regional offices, as well as by the Association of Football Coaches (GFT) from each of Bavaria’s administrative districts. The participating team coaches were informed about the methods of this study and the questionnaire. Coaches who did not complete the questionnaire in full were excluded from this study.

The online questionnaire, which analysed the prevalence of pain, the use of painkillers in amateur football players, the availability of painkillers, and the indication of painkiller use in detail, consisted of 45 items on different topics. Since coach recommendations can influence player painkiller use, it was analysed how much the coaches knew about the painkiller use in their teams and how often advice was given. Another important point, which was analysed, was the access to painkillers in the locker room. The questionnaire was based on the consensus statement on injury definitions and data collection procedures of Fuller et al. [13], as well as on the most common types of injuries in football [14], on the use of nutritional supplements, and the use of pain medication in football, as detailed in previous studies [15]. The most relevant items were summarized and adapted specifically for amateur football.

IBM SPSS Statistics Version 28.0 was used for the statistical analysis and graphical presentation of the tables and figures. For the descriptive presentation of the results, frequencies were used in a percentage in addition to absolute numbers.

The Ethics Committee of the University of Regensburg (No. 22-2781-101) approved this study.

## 3. Results

A total of 628 coaches from amateur football accepted the invitation to take part in this study, and 400 of them (63.7%) completed and submitted the questionnaire. In total, 369 male coaches and 31 female coaches were included in the survey in the end. Almost 95% of the participating team coaches were former football players. In terms of coaching licenses, more than 85% of the participants owned the official UEFA-B or C-license. Moreover, 12.7% of the coaches worked in salaried amateur football from the fourth to the seventh playing level in Germany, while 87.3% of them worked in real recreational amateur football (Table 1).

Overall, the team coaches stated that 36.2% of the players in their current teams already used painkillers for football-associated pain (SD = 29.2). The majority (84.7%) of them reported about football-associated pain in their teams over the entire season, and 60% of them reported football-associated pain occurring every month. The fact that football playing may be painful was shown by the coaches’ own previous injury histories, where just 0.5% of team coaches had no injury in their own career. The team coaches reported that 56.2% of them had used painkillers for football-related pain during their own playing careers (Table 2).

The availability of painkillers in amateur football showed that 85% of the coaches had received painkillers prescribed by a doctor for medically justifiable reasons. Additionally, 64% had bought over-the-counter painkillers at the pharmacy without a prescription. In contrast to this high availability, only around 10% of coaches left freely available painkillers for their players in the dressing room (Table 3). 

Around 75% of amateur team coaches saw no compatibility between painkillers and football competition, while a minority (around 25%) saw compatibility. Similarly, only a minority of around 7% of coaches would recommend the use of painkillers to maintain readiness to play, while 45% would never do so. According to the coaches, most pain medication was only taken after acute injuries or complaints in football (71.6%). However, 46% of coaches also stated that painkillers were sometimes taken preventively before a game (Table 4).

There were 80% of male team coaches who were in favour of the free availability of painkillers, while only 47.6% of their female colleagues said the same. Furthermore, 61.9% of the female coaches reported occasional or more frequent sport-independent pain, while only 47.6% of male coaches did so.

Significant age-related differences showed that team coaches over the age of 50 were more critical of the compatibility of painkillers and training compared to younger team coaches (over 50 years: 50%, under 32 years: 30%). It was also shown that coaches were rarely involved in the decision to take painkillers, regardless of age.

Around two thirds of the team coaches in the higher amateur football leagues (fourth division) saw a strong compatibility of painkillers with competition, while only around 20% of coaches in the lowest leagues saw this as the case. Painkillers were also available in around 80% of the first aid kits in the fourth division and around only 40% in the lowest leagues (Table 5). Due to the small number of participants from the fourth league, the differences in the league comparison were not statistically significant.

## 4. Discussion

The primary objective of this study, i.e., to analyse the use of painkillers in amateur football from coach perspectives, can be answered, for the first time, with a lifetime prevalence over the coaches’ own football careers of 36.2%, which is a lower rate when compared to comparable studies of professional football and other sports [3,15,16,17]. Some previous studies have described prevalences of painkiller use being over 90%; however, these numbers were recorded using different methods like doping control forms and, therefore, a direct comparison cannot be made in a sustainable manner [3]. Additionally, since the coaches, not the players, were surveyed in this study, it may have caused selection bias and led to a lower prevalence.

In total, 75% of the coaches in this study considered painkillers incompatible or rarely compatible with competition. This makes clear that the majority of the coaches in this study did not urge athletes to take painkillers and subordinate their health to the team’s sporting performance goals, as is often criticized in the literature [18,19]. Thus, a minority of team coaches reported that there were freely available painkillers in the dressing rooms; in particular, in the higher leagues, there was also a high prevalence of painkillers in the first aid kits. It was also found that 25% of all the coaches considered painkillers and competition to be at least predominantly compatible. This study is the first to describe this fact, which is the key point that will help to support future preventive strategies.

The data from this study also show that playing football is highly associated with pain during or after sports performance. Around 10% of the team coaches reported football-associated pain every match day or training session, and just under a quarter reported it once a week. These results are consistent with the data from previous literature. In a recent study from Sweden, in which over 500 amateur footballers were examined by means of a questionnaire regarding their football-related pain, a football-associated pain prevalence of around 20% per week was also reported [20]. The fact that acute injuries and overuse complaints are the most common reason for taking painkillers is again shown by the data from this study. Just over 70% of the team coaches stated that acute injuries are a reason for taking painkillers, and it was by far the largest group of occasions for taking painkillers in this study population. More problematic, however, was the fact that around 45% of coaches also stated that painkillers are occasionally taken prophylactically before matches. This has already been shown in other sports [21], and the results of this study make it clear that there is still a need for education regarding painkillers use that is not medically indicated, meaning there is potential for improvement.

It is also known that the role of coaches is very important for players and can influence player behaviour with regard to painkiller use [22]. However, coaches are usually only asked about this topic sporadically or not at all in this population. Only 8% of coaches are regularly involved with their players in their painkiller use. Regarding the coaching staff surveyed, only few coaches were found to directly influence the painkiller use of their players. Furthermore, the management style of the team and the attitude towards painkillers can also have an influence on the team [22]. It was a minority, but around 5% of the coaches approved the use of painkillers during or before important games. A study of comparable parameters could not be found in the literature, but this seems reason enough to promote more education and information in this regard such that as many coaches as possible can be reached. The data show that responsible use of painkillers and a solid understanding of potential side effects should be part of player and coach educations.

The availability of painkillers in the club was also recorded as easy access could increase use. Painkiller availability in first aid kits was reported by around 60% of coaches. The access also varied with playing level as there was a much higher availability in the highest analysed league (77.8%). It can also be claimed that the coaches were very responsible with the access to painkillers for their team as it was stated that, in over 90% of cases, medication was not left unattended in the changing room. The consideration of these environmental conditions, as in the present study, could not be found in the literature. Nevertheless, we saw that almost two thirds of the team coaches had already purchased over-the-counter painkillers for football-related pain without receiving a prescription.

When analysing the use of painkillers from the coaches’ point of view, this study also revealed differences in the subgroups that had not been described at all in the previous scientific literature. A major difference between the sexes was also evident in the opinion on the free sale of common painkillers. In this study, only 47.6% of the female coaches were in favour of continued free sale, while this same sentiment was significantly more common among the male coaches (80%). Female coaches in this study reported non-sport-related pain more frequently (61.9% vs. 47.6%). Similar sex-specific differences in pain frequency and painkiller use have also been noted in the literature. For instance, Hager et al. (2021) found a higher frequency of pain among female volleyball players compared to males, and Wezenberg et al. (2023) also demonstrated this for football-related pain [20,23]. The higher use of painkillers by women is not limited to sports as studies of the general population also show this trend, and comorbidities such as dysmenorrhea, psychosocial circumstances, and stereotypical gender roles can contribute to differences in pain expression [24,25].

Furthermore, the older coaches were much more critical of the use of painkillers than their younger colleagues. Accordingly, 50% of older coaches also considered the use of medication during their players’ training to be wrong, and 36% saw no occasion justifying the use of painkillers in football. Among younger coaches, this figure was only 33%. The players asked the older coaches, who were more critical of painkillers, for advice on painkiller use more frequently than the younger coaches. In total, 32% of the older coaches stated that they were occasionally or frequently asked for advice regarding painkillers and their use, while only 17% of the younger coaches group stated this. Data showing such age-related differences in coach attitudes to medication use have not been previously found in the literature and they were addressed for the first time in this study. 

The differences in the painkiller analysis with regard to different league levels showed that coaches in the lower leagues considered the compatibility of painkiller use and football to be significantly less compatible than coaches in the fourth league (22% vs. 66%). This is consistent with the data from the literature, where the higher performance levels also showed a higher use of painkillers in football, as well as in other sports [8,12,26,27]. However, the available data suggest that a higher level of performance generally correlates with an increased use of painkillers or a higher availability of painkillers. Increased sporting intensity and a higher level of training effort, as well as the resulting increase in pain and injuries, can explain this. The increased pressure, such as the players’ own ambitions and the expectations of the sporting environment, especially in the transition to the semi-professional sector, can also be a reason for higher painkiller use. It is particularly important to educate all the players involved about the use of painkillers, as well as their possible negative consequences for the players and also, therefore, for the teams. This can further improve the prevention of unindicated painkiller use [28].

Due to the online questionnaire-based prospective cohort study design, there were some limitations. First, investigating topics like painkiller use, which may be influenced by social desirability, poses challenges in verifying the authenticity of the responding coaches’ answers. There was also a possible selection bias in the separate analysis of the coaches. Furthermore, the participants were recruited exclusively from the federal state of Bavaria as the intensive cooperation between the Bavarian Football Association and its coaching associations made such a study possible. However, even though there are some methodological weaknesses of this study, there are no apparent reasons for the results not being transferable to the whole of German amateur football. The comparability of the subgroups may also be limited due to the fact that the population studied differed greatly in terms of sex and playing levels. The results of the current status of the painkiller prevalence in German amateur football from the perspective of coaches are the only ones in the literature to date, which makes it very difficult to classify them in the context of the literature. However, the fact that these initial results were presented with a large study population of 400 coaches provides a solid basis of data for further study projects on this topic. The large number of test subjects is a major strength of this study, particularly given the lack of data in amateur sports and the often poor response rates in the studies in this area.

## 5. Conclusions

This is the first study with information about painkiller use in amateur football from the perspective of team coaches. It can be shown that the prevalence of painkiller use in this study was significantly lower than was found in the data from the existing literature. The majority of coaches see the use of painkillers during games and training as incompatible, and only 10% indicated that painkillers were freely accessible in the dressing room. This study also shows that football is painful and that a large proportion of team coaches are already taking over-the-counter painkillers, which are freely available without a prescription.

For future educational work, the information of our study is essential. The importance of well-founded advice for players and coaches regarding injury breaks and the avoidance of painkillers in training or matches must be ensured, and a lively exchange between players and coaches in this regard must be promoted. Future research should also approach amateur football players directly in order to analyse their intrinsic urge to use painkillers in football.

## Figures and Tables

**Table 1:** The anthropometric and football-specific data of the population.

	Mean ± SD
Age in years	45.6 ± 11.9
Height in cm	183.6 ± 81.6
Weight in kg	85.7 ± 13.6
Sex = male, *n* (%)	369 (92.3%)
Coaching career in years	14.5 ± 10.3
Former football player, *n* (%)	369 (92.3%)
Paid amateur football, *n* (%)	42 (12.7%)
Pure amateur football, *n* (%)	278 (87.3%)
Coaching license, *n* (%) - UEFA A-license - UEFA B-license - UEFA C-license - Elite youth license - License precursors	9 (2.4%) 137 (36.5%) 198 (52.8%) 10 (2.7%) 21 (5.6%)

**Table 2:** The frequency of football-associated pain or injuries and the painkiller use in the team coaches’ careers.

Football-Associated Pain, *n* (%)	
Every match and training	46 (11.6%)
Once a week	95 (24.3%)
Once a month	96 (24.6%)
Once every six months	94 (24.0%)
Not at all	60 (15.3%)
Football-associated painkiller use in their own career, *n* (%)	
Already used once	218 (56.2%)
Never used	170 (43.8%)
Football-associated injuries during their career, *n* (%)	
Contusion	305 (77.4%)
Ligament injury	278 (70.6%)
Muscle strain	249 (63.2%)
Skin injury (abrasion/laceration)	224 (56.9%)
Muscle fibre tear	205 (52.0%)
Back pain	186 (47.2%)
Cartilage/meniscus injury	170 (43.1%)
Capsule injury	157 (39.8%)
Groin/hip pain	157 (39.8%)
Foot/heel pain	130 (33.0%)
Tendon pain (patella/Achilles tendon)	121 (30.7%)
Fracture	107 (27.2%)
Disc/nerve pain	67 (17.0%)
Muscle bundle rupture	64 (16.2%)
Concussion	60 (15.2%)
Other	41 (10.4%)
None	2 (0.5%)

**Table 3:** The availability of painkillers for amateur football players.

Medically Prescribed Painkillers, *n* (%)	
Have ever received	337 (85.5%)
Never received	57 (14.5%)
Painkillers purchased over-the-counter, *n* (%)	
Have ever acquired	251 (64.0%)
Never acquired	141 (36.0%)
Availability in the dressing room, *n* (%)	
Freely available	41 (10.6%)
Not available	347 (89.4%)

**Table 4:** The team coaches’ views on the compatibility of painkillers and football.

Compatibility of Painkillers and Competition, *n* (%)	
Fully compatible	4 (1.0%)
Very compatible	16 (4.1%)
Predominantly compatible	82 (20.9%)
Predominantly incompatible	194 (49.5%)
Not compatible	96 (24.5%)
Use of painkillers to maintain readiness to play, *n* (%)	
Many cases	7 (1.8%)
Occasionally	21 (5.4%)
In exceptional cases	185 (47.4%)
Never	177 (45.4%)
Reasons for painkiller use, *n* (%)	
Injuries	156 (71.6%)
Preventive before match	102 (46.8%)
Preventive before training	28 (12.8%)
General everyday life	36 (16.5%)
Not specified	2 (0.9%)

**Table 5:** The differences in the occurrence of pain and painkiller use between age groups and divisions.

Compatibility of Painkillers and Training, *n* (%)	Age under 32	Age over 50
Predominantly compatible	5 (9.8%)	10 (6.4%)
Predominantly incompatible	29 (56.9%)	68 (43.3%)
Not compatible	17 (33.3%)	79 * (50.3%) *p* = 0.047
Request for advice on painkillers, *n* (%)		
Always	0 (0.0%)	1 (0.6%)
Often	1 (2.0%)	16 (10.3%)
Occasionally	8 (15.7%)	33 (21.2%)
Rare	22 (43.1%)	57 (36.5%)
Never	20 (39.2%)	49 (31.4%)
Compatibility of painkillers and competition, *n* (%)	4th league	Lowest 5 leagues
Very compatible	1 (11.1%)	9 (3.8%)
Predominantly compatible	5 (55.6%)	46 (19.4%)
Predominantly incompatible	1 (11.1%)	126 (52.9%)
Not compatible	2 (22.2%)	57 (23.9%)
Availability of painkillers in the first aid kit, *n* (%)		
Available	7 (77.8%)	89 (37.4%)
Not available	2 (22.2%)	149 (62.6%)

* *p* < 0.05.

## Data Availability

The data generated in this study are included in the results of the published article.
